# 

**Published:** 1898-11-19

**Authors:** 


**Notices of Births, Deaths, and Marriages are inserted in this column at a charge of 2s. 6d. for an announcement not exceeding four lines.**

### NOTICE TO CORRESPONDENTS.

All MS., Letters, Books for Review, and other matters intended for the Editor should be addressed The Editor, "The Hospital," The Hospital Building, 28 & 29, Southampton Street, Strand, W.C.
The Editor cannot undertake to return rejected MS., even when accompanied by stamped directed envelope.

**Notice to Advertisers and Subscribers.**
All Advertisements, Orders for Copies of The Hospital, and Business Communications should be addressed to **THE MANAGER (Not to the Editor),** The Hospital, The Hospital Building, 28 & 29, Southampton Street, Strand, London, W.C.
The Cost of Subscription, post free, is as follows:—Per week, 2½d.; per quarter, 2s. 8d.; per half-year, 5s. 3d.; per annum, 10s. 6d. Foreign:—Per annum, 15s. 2d., payable, in all cases, in advance.

**NOTICE.—Vol. XXIV. of "The Hospital" (April, 1898, to September, 1898), in handsome cloth binding, is now on sale at the Office of this Journal, price 7s. 6d. Cases for binding the Numbers contained in this Volume 1s. 6d. Index to Vol. XXIV., 2½d., post free.**

**The Monthly Part for November is now ready. Price 6d., by post 8d.**

### Scraps and Gleanings.

The doctors of the Forfar Infirmary are to have their honorarium increased from £15 to £20 per annum.

The Middlesex Hospital has received a contribution of £100, being the first yearly instalment of a donation of £500 from the Worshipful Company of Skinners.

The bazaar held at Rugby to complete the erection and furnishing of the new Diamond Jubilee Wing at the Hospital of St. Cross has realised a sum of about £700.

The work in connection with the establishment of proper accident wards and operating-room at the Devonshire Hospital and Buxton Bath Charity has now been commenced.

The one hundred and twenty-eighth anniversary of the Radcliffe Infirmary, Oxford, and the thirty-third of the dedication of the chapel, was kept on Tuesday, October 18th.

Dr. Frank Renaud, the consulting physician to the Manchester Royal Infirmary, has compiled a volume, entitled "A Short History of the Manchester Royal Infirmary from 1752 to 1877."

A Parish Association in aid of the Aberdeen Royal Infirmary has been formed at Drumoak. The appeal on behalf of the infirmary is to be made direct to the people, and not through the Churches.

The cost of the new out-patients' department at the Ancoats Hospital has been estimated at £6,000, a sum which the children of the late honorary treasurer have most generously offered to provide. The building operations have already been begun.

A sub-committee has been appointed by the managers of the Huntly Cottage Hospital to represent them at a joint meeting of the committees appointed by the local authorities to consider the question of providing hospital accommodation for infectious diseases.

The Committee of Management of the Mercy Hospital are making an appeal on behalf of the institution under their charge in order to liquidate the debt of £500 which the management have been compelled to incur in their effort to maintain the usefulness of the hospital.

In connection with the centenary celebration of the Plymouth Public Dispensary, it has been suggested that certain structural alterations should be made to improve the accommodation in the physicians' department, and the House Committee have been requested to investigate the subject.

The relief given during the past year at the Ham Yard Soup Kitchen and Hospice, Great Windmill Street, Piccadilly Circus, W., reached the total of 236,281 meals. Of these 91,079 were penny dinners to boardmen, the rest free to families. The hospice afforded 5,127 free nights' lodgings to destitute men, many of whom found work during their stay. A Christmas dinner to 1,030 poor families and 160 tons of coal were also distributed.

### THE STUDENT OF MEDICINE.

### APPOINTMENTS.

B. J. ADAM, M.B.Glasg., appointed Health Officer for the Borough of Daylesford, Victoria. S. E. Atkins, L.R.C.S.I. L.S.A., appointed Medical Officer for the Hatherleigh District of the Okehampton Union. W. B. Bennett, M.R.C.S., L.R.C.P. Lond., appointed Assistant Medical Officer to the Parish of Liverpool Workhouse. G. Bill, M.B.Edin., appointed Public Vaccinator at Pyramid Hill, Victoria, and Health Officer for East Loddonshire, East Riding, Victoria. Anna L. Church, M.D. (R.U.I.), appointed Medical Officer in charge of the Victoria Hospital, Calcutta. J. W. E. Cole, M.R.C.S.Eng., appointed Assistant Medical Officer at the Bow Infirmary of the City of London Union. G. E. Crowe, B.A.Dub., M.D., appointed Medical Officer to the Cottage Homes of the Chorlton Union. T. B. P. Davies, M.D., M.S.Lond., M.R.C.S., L.R.C.P., appointed Surgeon to the Criterion Gold Mining Company, Bulawayo. C. F. Druitt, M.R.C.S., L.R.C.P., appointed House Surgeon to the Paddington Green Children's Hospital. R. C. Field, M.B.Lond., appointed Resident Obstetric Officer to the General Infirmary, Leeds. J. F. R. Gairdner, M.B., C.M. Glasg., M.R.C.S.Eng., L.R.C.P.Lond., appointed Resident Surgical Officer to the General Infirmary, Leeds. H. E. Goulden, M.R.C.S., L.R.C.P., D.P.H.Camb., appointed House Physician to the Paddington Green Children's Hospital. J. Gregg, M.B., B.S.Dub., appointed Public Vaccinator at Wedderburn, Victoria. J. M. Hermon, M.B., C.M.Edin., appointed Medical Officer for the Crofton District of the Wakefield Union. H. H. I. Hitchon, L.R.C.P.Lond., M.R.C.S., appointed Medical Officer of Health for the Borough of Heywood. W. M. Hutton, M.D., F.R.C.S. Edin., appointed Surgical Registrar to the Royal Infirmary, Edinburgh. J. B. Johnson, M.D., C.M.Montreal, L.S.A., appointed Medical Officer for the Lavenham District of the Cosford Union. W. D. Loveday, M.R.C.S., L.R.C.P.Lond., appointed Medical Officer for the Hendred District of the Wantage Union. S. McDonald, M.B., C.M., appointed Pathologist to the General Hospital, Birmingham. H. C. Major, M.D.Edin., appointed a Consulting Physician to the Bradford Royal Infirmary. J. Martin, L.R.C.P., L.R.C.S.Edin., appointed Health Officer for the Broad Arrow, West Australia. H. O. Moore, M.B., appointed Health Officer for the Southern Portion of Karkaroocshire and Public Vaccinator for Beulah, Victoria. W. F. Oyston, M.B., Ch.B.Vict., appointed House Physician to the General Infirmary, Leeds. G. E. Scholefield, M.D., D.P.H., appointed Medical Officer of Health to the West Lancashire Rural District Council. W. P. Seed, M.R.C.S., F.R.C.P.Lond., appointed Resident Medical Officer and Public Vaccinator for Coolgardie, Western Australia. J. H. Sequeira, M.D.Lond., F.R.C.S.Eng., appointed Physician to the Tower Hamlets Dispensary. H. Smith, M.A., M.B., B.C.Cantab., appointed Honorary Physician to the Boscombe Hospital, Bournemouth. A. Spong, M.B, Ch.B.Vict., appointed Resident Medical Officer at the Ida Hospital, Leeds. H. Stansfield, M.B., Ch.B.Vict., appointed House Physician to the General Infirmary, Leeds. E. Trotter, M.B., Ch.B.Vict., appointed House Surgeon to the General Infirmary, Leeds. E. Turton, M.B., Ch B.Vict., appointed House Surgeon to the General Infirmary, Leeds. F. J. Walden, M.B., C.M.Edin., L.M.Rotunda Hos. Dub., appointed Medical Officer for the Sorell District, Tasmania.

NOW READY,

Demy 8vo., green buckram, gilt, 7s. 6d. net.

### AN ATLAS OF BACTERIOLOGY.

Containing 111 Original Photo-micrographs, with Explanatory Text.

By CHARLES SLATER, M.A., M.B., M.R.C.S.Eng., F.C.S.; and EDMUND J. SPITTA, L.R.C.P.Lond., M.R.C.S.Eng, F.R.A.S.

"The book contains one hundred and eleven photo-micrographs, which exceed in clearness anything of the kind that has yet been published. It may safely be said that the 'Atlas' will be found indispensable by every practical bacteriologist."—*Pharmaceutical Journal.*

London: THE SCIENTIFIC PRESS, LTD., 28 & 29, SOUTHAMPTON STREET, STRAND, W.C.

BEFORE FURNISHING YOUR HOSPITAL or CONVALESCENT HOME

WRITE TO, OR CALL UPON

### R. & W. WILSON & SONS, LIMITED,

**90, WARDOUR STREET, LONDON, W.,**

For: IRONMONGERY, COPPER, BRASS, AND TIN WARE OF EVERY DESCRIPTION; CUTLERY, BRUSHES AND BROOMS, ENAMEL WARE, &c.; SPECIALITIES IN HOSPITAL REQUISITES.

*ESTIMATES GIVEN for Furnishing Convalescent Homes and similar Institutions.*

### WORKS FOR NURSES.

**A MANUAL FOR HOSPITAL NURSES** AND OTHERS ENGAGED IN ATTENDING ON THE SICK. By EDWARD J. DOMVILLE, L.R.C.P., M.R.C.S. Eighth Edition. Crown 8vo., 2s. 6d.

**A MANUAL OF NURSING, MEDICAL** AND SURGICAL. By C. J. CULLINGWORTH, M.D., F.R.C.P. Third Edition. Fcap. 8vo., 2s. 6d.

**A SHORT MANUAL FOR MONTHLY** NURSES. By C. J. CULLINGWORTH, M.D. Fourth Edition. Revised by M. A. ATKINSON. Fcap. 8vo., 1s. 6d.

**NOTES ON GYNÆCOLOGICAL NURSING.** By J. B. HELLIER, M.D. Fcap. 8vo., 1s. 6d.

**HINTS ON ELEMENTARY PHYSIOLOGY.** By FLORENCE A. HAIG-BROWN. With a Preface by Dr. ORD. With 21 Illustrations. Fcap. 8vo., 1s. 6d.

**LECTURES ON MEDICINE TO NURSES.** By HERBERT E. CUFF, M.D. Second Edition. With 29 Illustrations. Crown 8vo., 3s. 6d.

**ANTISEPTIC PRINCIPLES FOR NURSES.** By C. E. RICHMOND, F.R.C.S. Fcap. 8vo., 1s.

**A SHORT DICTIONARY OF MEDICAL** TERMS. 2s. 6d.

**CHAVASSE'S ADVICE TO A MOTHER** ON THE MANAGEMENT OF HER CHILDREN, and on the Treatment on the Moment of their more Pressing Illnesses and Accidents. Fifteenth Edition by Dr. GEORGE CARPENTER, Physician to the Evelina Hospital for Sick Children. 2s. 6d.

**CHAVASSE'S ADVICE TO A WIFE ON** THE MANAGEMENT OF HER OWN HEALTH, and on the Treatment of Some of the Complaints incidental to Pregnancy, Labour, and Suckling. With an Introductory Chapter especially addressed to a Young Wife. Fourteenth Edition, revised by Dr. FANCOURT BARNES, Consulting Physician to the British Lying-in Hospital. 2s. 6d.

London: J. and A. CHURCHILL,

7, Great Marlborough Street.

